# Non-Glycosylated SARS-CoV-2 Omicron BA.5 Receptor Binding Domain (RBD) with a Native-like Conformation Induces a Robust Immune Response with Potent Neutralization in a Mouse Model

**DOI:** 10.3390/molecules29112676

**Published:** 2024-06-05

**Authors:** Rawiwan Wongnak, Subbaian Brindha, Mami Oba, Takahiro Yoshizue, Md. Din Islam, M. Monirul Islam, Hitoshi Takemae, Tetsuya Mizutani, Yutaka Kuroda

**Affiliations:** 1 Department of Biotechnology and Life Science, Faculty of Engineering, Tokyo University of Agriculture and Technology, Nakamachi 2-24-16, Tokyo 184-8588, Japan; s218131w@st.go.tuat.ac.jp (R.W.); brindha@m2.tuat.ac.jp (S.B.); jimoxianguang@gmail.com (T.Y.); s224333x@st.go.tuat.ac.jp (M.D.I.); 2 Institute of Global Innovation Research, Tokyo University of Agriculture and Technology, Tokyo 183-8538, Japan; mamioba@go.tuat.ac.jp (M.O.); fy7210@go.tuat.ac.jp (H.T.); tmizutan@cc.tuat.ac.jp (T.M.); 3 Center for Infectious Disease Epidemiology and Prevention Research, Tokyo University of Agriculture and Technology, 3-5-8 Saiwai-Cho, Fuchu-shi 183-8509, Japan; 4 Department of Biochemistry and Molecular Biology, Faculty of Biological Sciences, University of Chittagong, Chittagong 4331, Bangladesh; islammm@cu.ac.bd

**Keywords:** Omicron SARS-CoV-2, neutralization, non-glycosylation, molten globule state, native-like state, immunogenicity, *E. coli*-expression system, disulfide bonds, limited proteolysis

## Abstract

The Omicron BA.5 variant of SARS-CoV-2 is known for its high transmissibility and its capacity to evade immunity provided by vaccine protection against the (original) Wuhan strain. In our prior research, we successfully produced the receptor-binding domain (RBD) of the SARS-CoV-2 spike protein in an *E. coli* expression system. Extensive biophysical characterization indicated that, even without glycosylation, the RBD maintained native-like conformational and biophysical properties. The current study explores the immunogenicity and neutralization capacity of the *E. coli*-expressed Omicron BA.5 RBD using a mouse model. Administration of three doses of the RBD without any adjuvant elicited high titer antisera of up to 7.3 × 10^5^ and up to 1.6 × 10^6^ after a booster shot. Immunization with RBD notably enhanced the population of CD44^+^CD62L^+^ T cells, indicating the generation of T cell memory. The in vitro assays demonstrated the antisera’s protective efficacy through significant inhibition of the interaction between SARS-CoV-2 and its human receptor, ACE2, and through potent neutralization of a pseudovirus. These findings underscore the potential of our *E. coli*-expressed RBD as a viable vaccine candidate against the Omicron variant of SARS-CoV-2.

## 1. Introduction

The Omicron variant of SARS-CoV-2 has rapidly become the predominant circulating strain shortly after its emergence. Unlike other strains that predominantly target the lungs, the Omicron variant primarily infects cells in the upper respiratory tract, which have fewer ACE2 receptors. This results in higher infectivity, potentially due to an enhanced binding affinity to the ACE2 receptor compared to the original Wuhan strain [1].

In particular, the BA.5 subvariant, which has 17 mutations within the receptor-binding domain (RBD) from original strains, exhibits increased transmissibility and a greater ability to evade immune responses compared to its predecessors [2,3]. For instance, mutations such as L452R and F486V within the RBD are identified as crucial for immune evasion [4]. Accordingly, the BA.5 variant has a higher rate of infection in vaccinated and COVID-19-recovered individuals [5,6]. Furthermore, the presence of mutations in the Omicron BA.5 variant has been associated with a notable reduction in the efficacy of therapeutic monoclonal antibodies [7], underscoring the urgent need for vaccines specifically designed to target the Omicron variant’s unique epitopes to maintain adequate protection against SARS-CoV-2 [8].

In addition to its increased infectivity and immune evasion capabilities, the BA.5 variant has also been linked to changes in clinical COVID-19 manifestation. While it appears to cause less severe symptoms than some previous variants, particularly in vaccinated individuals, but it can still lead to significant morbidity and stress on healthcare systems due to its high transmissibility [9]. Symptoms associated with BA.5 infections often include sore throat, cough, fatigue, and nasal congestion, but severe outcomes can still occur, especially in vulnerable populations such as the elderly and those with underlying health conditions [10,11].

Subunit vaccines offer several advantages in the context of SARS-CoV-2 vaccination. They comprise specific protein subunits or fragments derived from the virus rather than the whole pathogen [12]. This targeted approach allows for selecting immunogenic components to induce a robust immune response while minimizing the risk of adverse reactions associated with whole pathogen vaccines [13]. Moreover, compared to other modalities such as mRNA vaccines, subunit vaccines are made of protein, which is generally stored at refrigeration temperatures and does not need ultra-cold storage, simplifying storage and transportation logistics [14].

**Figure 1:**
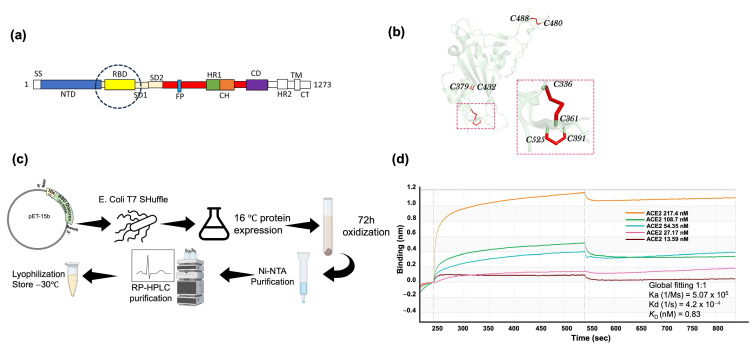
SARS-CoV-2 Omicron BA.5 RBD expression and purification in *E. coli*. (**a**) Schematics of the sequence location of RBD in the SARS-CoV-2 spike protein. (**b**) Ribbon model of SARS-CoV-2 RBD with disulfide bond pairing. (**c**) SARS-CoV-2 Omicron BA.5 RBD expression and purification protocol in *E. coli*. (**d**) Binding of the SARS-CoV-2 Omicron BA.5 RBD to the hACE2 using an Octet-N1 Bio-Layer Interferometer. RBD was immobilized on a Ni-NTA sensor chip, and hACE2 was in the mobile phase.

The RBD-spanning residues Arg319–Phe541 of the spike protein (Figure 1a) is a relatively small 25 kDa beta-sheeted protein, containing eight cysteines forming four disulfide bonds (Figure 1b) [15]. RBD is a promising vaccine target against SARS-CoV-2 due to its critical role in viral entry into the host cell. It is responsible for binding to the angiotensin-converting enzyme 2 (ACE2) receptor on host cells, facilitating viral entry [16]. Targeting the RBD can prevent this interaction, thereby blocking viral entry and subsequent infection. Moreover, the RBD contains epitopes that can potentially stimulate a robust immune response through the production of neutralizing antibodies. Vaccines targeting the RBD have demonstrated the ability to induce high levels of neutralizing antibodies [17], essential for blocking viral infection [18,19]. However, recent investigations, including computational modeling and experimental approaches, suggest that the Omicron RBD may exhibit lower immunogenicity than the wild-type strain [20]. Consistent with these findings, research on recombinant protein vaccines suggests that the Omicron RBD-produced antisera exhibits a diminished neutralization against the original strain [21]. These reports thus suggest that one needs to adequately control the immunogenicity of the Omicron S1 protein, or its RBD region, to develop a vaccine targeting this variant.

Bacterial, especially *E. coli*, expression systems present compelling advantages over insect and mammalian cell systems in terms of cost, yield, and production time [22,23]. However, *E. coli* expression systems lack post-translational processing mechanisms and standard expression protocols produce non-glycosylated proteins, and in many instances misfolded proteins forming non-native SS bonds. In a previous study, we optimized the *E. coli* expression protocol to produce large quantities of RBD, forming four native SS bonds and having native-like structural and biophysical properties [24,25]. In the present study, we report that the native-like folded *E. coli*-produced non-glycosylated Omicron RBD elicited a robust immune response with long-term immune memory. Further, it produced neutralizing antisera in mouse models, despite the lack of glycosylation, as assessed using pseudoviruses. These promising outcomes underscore the potential of our approach as a candidate for a SARS-CoV-2 vaccine. Additionally, the potent immunogenicity and neutralizing ability of the *E. coli*-expressed RBD could provide valuable insights for optimizing vaccines with enhanced protection against SARS-CoV-2.

## 2. Results

### 2.1. SARS-CoV-2 Omicron BA.5 RBD Expression and Purification in E. coli and Biochemical Characterization

We expressed SARS-CoV-2 Omicron BA.5 RBD utilizing the *E. coli* T7 SHuffle cell line, an engineered strain designed to optimize disulfide bond formation within the cytoplasm [26]. We eventually implemented a strategy of low-temperature induction to regulate production rates, thereby facilitating correct disulfide bond pairing [27]. Subsequently, further oxidation in GuHCl at pH 8.8 completed the formation of disulfide bonds [28]. Further purification was conducted by denaturing Ni-NTA chromatography and RP-HPLC (Figure 1c). Building upon our prior study, which extensively characterized the *E. coli*-produced RBD, our findings revealed a native-like structural integrity characterized by a high thermal stability, native SS bonds, and a strong binding to the human SARS-CoV-2 receptor hACE2 [24]. Notably, the dissociation constant (*K*_D_) of RBD-hACE2 was 0.83 nM, calculated from the kinetic curves (Figure 1d), which is comparable to the binding of S1 expressed in mammalian systems [29]. These observations corroborate the native-like nature of RBD expressed in *E. coli* and, in particular, its native epitopes.

### 2.2. E. coli-Expressed RBD Induced a High Level of Anti-RBD Antisera in a Mouse Model

**Figure 2:**
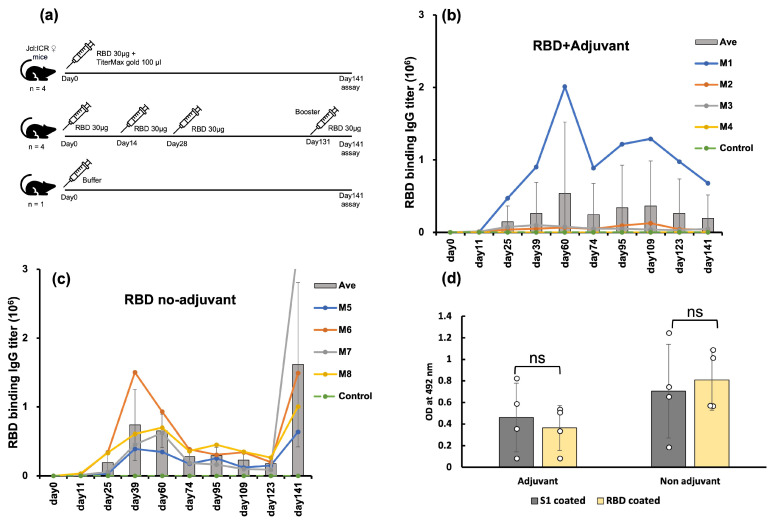
The immunogenicity of *E. coli*-expressed RBD in a mouse model. (**a**) Immunization scheme with adjuvant, no adjuvant, and control groups. (**b**) IgG titer assays by ELISA of RBD-immunized mice with adjuvant (*n* = 4). Individual mouse IgG titers are represented by circles, and the average titer is presented in bars. (**c**) IgG titer assays by ELISA of RBD-immunized mice without adjuvant (*n* = 4). Individual mouse IgG titers are represented by circles, and the average titer is represented with bars. (**d**) Recognition of mammalian-expressed S1 spike protein by *E. coli*-expressed RBD antisera (1:1000 dilution). Circles represent the OD at 492 nm of four mice from each group (adjuvant and no adjuvant), indicating the binding of antisera collected in the 9th week after the first administration. Measurements were taken using mammalian-expressed spike (S1) protein or *E. coli*-expressed RBD as coating antigens for comparison. The average OD value of each group is presented in bars. The analysis revealed no significant difference (*p* > 0.05) between the S1-coated and RBD-coated results, ‘ns’ stands for not significant.

To evaluate the immunogenicity of *E. coli*-expressed RBD, we conducted two separate sets of immunization experiments utilizing Jcl:ICR mice. One group received the RBD along with an adjuvant (TiterMax Gold) administered as a single dose. Given the adjuvant’s capacity to enhance immune responses in murine models, a single dose sufficed for this group [30]. Conversely, we opted for a three-dose regimen for the no adjuvant group; this mouse group received three consecutive doses of RBD only. The control mouse group was injected only with buffer (Figure 2a).

The anti-RBD antibody titers of three out of four mice in the adjuvanted group were high, while one mouse displayed a negligible response (Figure 2b). The antibody titers of these three mice peaked nine weeks after the injection. This slow response is often attributed to the ability of the adjuvant to deposit the antigen at the injection site and gradually release it, thereby prolonging the generation of an immune response [30,31]. Mouse 1 showed the highest antibody titer. Conversely, all four mice immunized with RBD without adjuvant exhibited a robust immune response ten days after the third injection, followed by a gradual decline (Figure 2c). To validate the memory response, a booster was administered to the non-adjuvanted group on day 131. The booster shot significantly increased the antibody titers by 9-fold, ten days after the booster shot. Thus, RBD induced a high immune response in mice even in the absence of an adjuvant, indicating the high potential of *E. coli*-expressed RBD as a vaccine antigen.

### 2.3. E. coli-Expressed RBD Elicited Antisera Recognized the Mammalian-Produced S1

We assessed the antisera’s ability to recognize the native, mammalian-expressed spike S1 protein using ELISA. The binding of antisera against the *E. coli*-expressed RBD was similar to that against the mammalian cell-expressed S1-protein (Figure 2d). This confirmed that the antisera raised against *E. coli*-expressed RBD targeted the native epitopes of the S1-protein and thus the native spike protein.

### 2.4. CD Marker Analysis through Flow Cytometry

**Figure 3:**
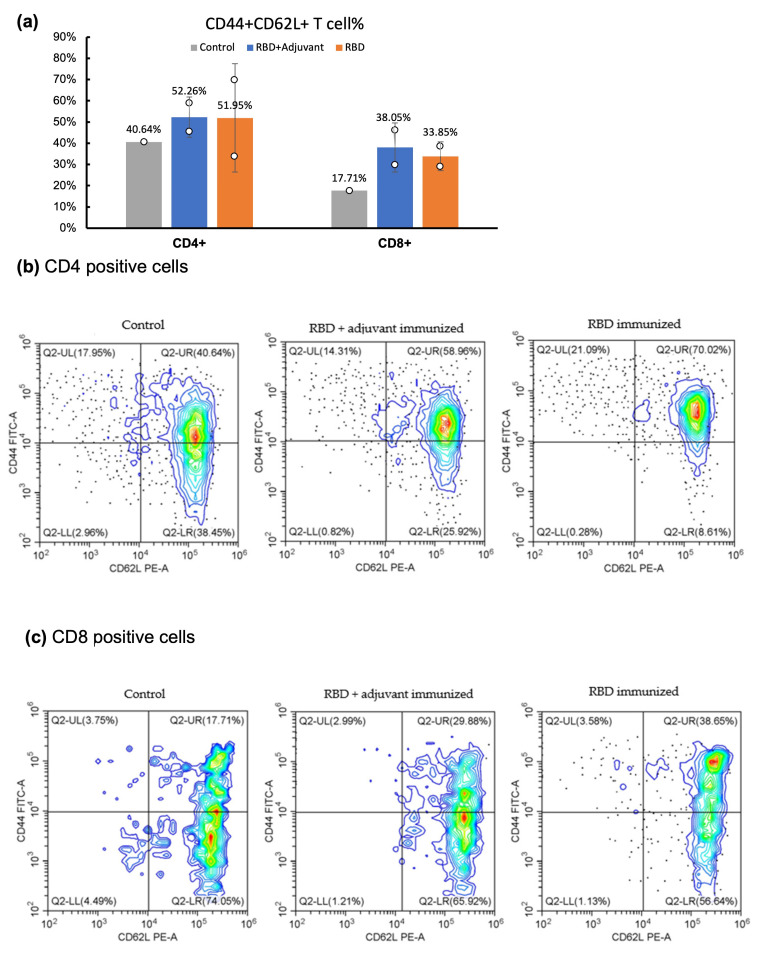
Effects of *E. coli*-expressed RBD immunization on T-cell memory assessed through CD marker analysis by flow cytometry. (**a**) The bar graph illustrates the percentage of CD44^+^CD62L^+^ T cells in the control mouse (*n* = 1) compared with the average percentage of CD44^+^CD62L^+^ T cells in two mice (*n* = 2) from the adjuvant and no adjuvant groups. The individual data are represented by circles. (**b**) Cluster of differentiation (CD) expression of CD4^+^ (T-helper cell) surface in the single mouse from each group. (**c**) Cluster of differentiation (CD) expression of CD8^+^ (T-cytolytic cell) surface in the single mouse from each group. Note: Due to the absence of multiple data points within the control group (*n* = 1), no statistical analysis was performed.

Central T-cell memory analysis was conducted using flow cytometry to examine cell surface markers. The T-cell response and long-term memory were assessed by analyzing CD (cluster of differentiation) markers in spleen samples collected 141 days after the initial injection. In contrast to the control mouse injected solely with buffer, both the adjuvant and the no adjuvant group exhibited increased average populations of CD44^+^CD62L^+^ cells in both CD4^+^ (T helper cells) and CD8^+^ (cytotoxic T cells) (Figure 3). This observation suggests that both groups generated more central T-cell memory than the control mouse, in terms of both CD4^+^ and CD8^+^ populations [32,33].

### 2.5. RBD-hACE2 Binding Inhibition Assay

**Figure 4:**
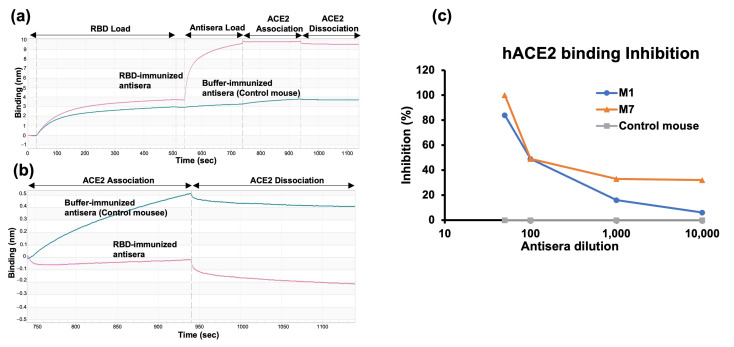
hACE2 inhibition assay. (**a**) Steps of the hACE2 binding inhibition assay using bio-layer interferometry (BLI). RBD was immobilized on the biosensor chip, followed by antisera binding. hACE2 was loaded for the association and dissociation steps assessed in the kinetic buffer. (**b**) Enlarged figure of hACE2 association and dissociation step. (**c**) Inhibition of RBD binding to hACE2 by *E. coli*-expressed RBD-immunized antisera of one mouse from each group; “M” indicates the identity of the mouse.

We first evaluated the neutralization potential of the antisera by assessing the binding inhibition of the RBD to hACE2. This measurement was conducted using antisera collected 141 days after the first injection from a single mouse in each group. The degree of inhibition of RBD binding to hACE2 was evaluated by bio-layer interferometry (BLI). The degree of inhibition was calculated with respect to the control mouse antisera, which exhibited no (0%) inhibition (Figure 4a,b). Antisera from the mouse immunized with RBD in the presence of adjuvant (M1) exhibited lower inhibition than antisera from the mouse immunized with RBD alone (M7). The mouse immunized with non-adjuvanted RBD produced antisera, achieving 100% inhibition at a 1:50 dilution (Figure 4c).

### 2.6. Pseudovirus Neutralization

**Figure 5:**
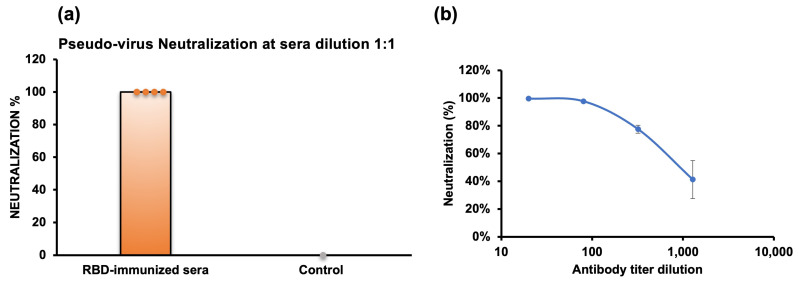
Pseudovirus neutralization assay. (**a**) Neutralization (%) of pseudovirus by antisera at 141 days after the first administration of RBD. Neutralization percentages of individual mice (*n* = 4) from the no adjuvant group are represented by circles, and the average is presented with bars. (**b**) Pseudovirus neutralization titers (ID_50_) analysis of a single mouse from the no adjuvant group (M7). The inhibition rate against pseudovirus is plotted against the reciprocal of the antisera dilution. Note: RBD-immunized data (*n* = 4) showed 100% neutralization with no variance (mean = 100%, standard deviation = 0%), while the single data point in the control group showed 0% neutralization. Due to the lack of variability in the RBD-immunized group, traditional statistical tests such as the *t*-test were not applicable. Descriptive statistics are provided to illustrate the results.

We further evaluated the neutralizing activity using a pseudovirus neutralization assay, employing antisera collected from a heart bleed ten days after the booster dose. The neutralizing antisera would target the RBD of the spike protein, hindering the binding of the pseudovirus, which carries the firefly luciferase reporter gene, to the ACE2 receptor. As a result, we observed that the antisera of all non-adjuvanted RBD-immunized mice completely neutralized the pseudovirus at a 1:1 dilution (Figure 5a), demonstrating that the antisera effectively inhibited pseudovirus infection. Conversely, the antisera from the RBD with adjuvant immunization group exhibited decreased neutralization, likely due to the lower antibody titers.

We further measured the 50% pseudovirus neutralization titer (ID_50_) of anti-RBD antisera from the highest-titer mouse in the no adjuvant group (M7) (Figure 5b). The ID_50_ was 1:901 dilution, calculated using the Quest Graph™ IC50 Calculator [34] (Supplementary Figure S1).

## 3. Discussion

The immunogenicity results of the *E. coli*-expressed receptor-binding domain (RBD) demonstrated that, even in the absence of an adjuvant, the *E. coli*-expressed RBD elicited a robust immune response, reaching titers as high as 7.3 × 10^5^ ten days after the administration of the third dose. In contrast, the group of mice immunized with the RBD in conjunction with an adjuvant did not uniformly exhibit a high immune response. This variability could be attributed to the differential sensitivity among individual mice or potentially to the insufficiency of a single injection of the RBD with adjuvant to induce a comparable immune response.

Our study focused on the *E. coli*-expressed Omicron BA.5 RBD, which we extensively characterized to confirm its native-like conformational and biophysical properties [24]. In particular, we demonstrated that the SS bonds are correctly formed, and a cooperative thermal denaturation monitored by circular dichroism strongly suggested that the overall conformation was native-like [35,36]. However, despite numerous attempts to optimize the measurement conditions, we did not observe sharp and well-dispersed peaks in the NMR spectra nor a strong heat absorption peak by differential scanning calorimetry (DSC; Dr. T. Saotome, *personal communication*) that are hallmarks of a “native” state [37]. We thus believe that RBD holds a naïve-like fold, but the structure is not as rigid as a native one and might be close to a molten globule state [38,39]. Nevertheless, the *E. coli*-expressed RBD elicited a robust immune response with neutralizing antisera, which is remarkable given the common belief that a native state is required for such a response. 

Furthermore, the strong immunogenicity of the *E. coli*-expressed RBD also demonstrates that glycosylation is not necessarily required for the production of neutralizing antisera. This would be in line with reports suggesting that the lack of glycosylation facilitates the efficient recognition and processing of antigens by antigen-presenting cells (APCs), contributing to a potent immune response [40,41]. Thus, despite its small size (25 kDa), RBD induced a robust immune response with high antibody titers, and a booster shot 102 days after the last immunization further increased the titer, strongly suggesting the development of long-term memory. Cell surface marker analysis confirmed this observation and indicated that RBD triggered the spleen cells to generate central memory T cells, a crucial component of long-lasting immunity. 

The SARS-CoV-2 Omicron infection inhibition potential of the antisera was, at first, assessed through hACE2 binding inhibition using bio-layer interferometry (BLI) experiments. In vitro assays demonstrated that the antisera elicited by Omicron RBD completely inhibited the binding of RBD to ACE2 receptors at a 1:50 dilution in mouse 7 from the no adjuvant group. Subsequent analysis via pseudovirus neutralization assays revealed a potent neutralizing ability of mouse antisera produced against *E. coli*-expressed Omicron RBD, effectively blocking pseudovirus infections in HEK cells, with all mice in the no adjuvant group showing 100% neutralization.

To date, ID_50_, indicative of the antibody concentration required to neutralize 50% of the virus, provides valuable insights into the potency of the antibody response and is crucial for assessing the efficacy of a vaccine seed. The ID_50_ value of our RBD was 1:901 dilutions and was comparable to that of Novavax, which is an approved anti-SARS-CoV-2 subunit vaccine [39]. Other approved vaccines against SARS-CoV-2 show ID_50_ values between 1000 and 10,000 [42,43]. The high neutralization of the antisera produced by our *E. coli*-expressed RBD is in line with the rationale that the antisera can bind to unmasked epitopes due to the non-glycosylation of proteins expressed in *E. coli* [44,45,46]. Overall, our results emphasize that an *E. coli*-expression system can be used to produce small proteins eliciting the production of neutralizing antisera, thereby underscoring its suitability for protein expression in vaccine development.

## 4. Materials and Methods

### 4.1. Protein Expression and Purification

Protein expression and purification were described in detail in our previous report [25,47]. In short, a DNA sequence encoding SARS-CoV2 RBD Omicron BA.5 with a His-tag was inserted into the pET15b vector. The recombinant plasmid was introduced into *E. coli* T7 SHuffle cells (New England Biolabs, Ipswich, MA, USA) through transformation. The transformed cells were initially cultured overnight at 30 °C with shaking at 250 rpm in 5 mL of LB medium containing 50 μg/mL ampicillin. Subsequently, this pre-culture was transferred into 200 mL of LB medium with the appropriate antibiotics and incubated at 30 °C with shaking until the optical density at 600 nm (OD600) reached approximately 0.6. Gene expression was then induced by adding 0.25 mM IPTG, followed by incubation at 16 °C with shaking at 250 rpm for 16–18 h. Cells were harvested by centrifugation at 8000 rpm using a Hitachi himac CF16RX centrifuge with a T9A31 rotor (Hitachi, Tokyo, Japan) at 4 °C for 20 min. The cell pellets were resuspended in lysis buffer (50 mM Tris-HCl pH 8, 150 mM NaCl) and disrupted by sonication in a buffer containing 50 mM Tris-HCl pH 8, 1% NP-40 (*v*/*v*), 0.1% deoxycholic acid (*w*/*v*), and 5 mM EDTA. Inclusion bodies were collected by centrifugation at 8000 rpm for 20 min.

The inclusion bodies were then solubilized and subjected to air oxidation for 72 h at 25 °C in 6 M guanidine hydrochloride with 50 mM Tris-HCl, pH 8.8. After centrifugation at 8000 rpm at 4 °C for 20 min and filtration through a 0.2 µm filter, the 6× histidine-tagged protein was purified using denaturing nickel-nitrilotriacetic acid (Ni-NTA) chromatography (Wako, Tokyo, Japan). The column was washed three times with wash buffer (6 M GuHCl, 50 mM Tris-HCl pH 6.8), and proteins were eluted with elution buffer (6 M GuHCl and 10% acetic acid). Guanidine hydrochloride was removed by dialysis against reverse osmosis (RO) water for 18 h at 4 °C with four exchanges of the outer solution, using a dialysis membrane with a molecular weight cut-off (MWCO) of 14,000. The sample was then centrifuged at 8000 rpm at 4 °C for 20 min, and the supernatant was separated from the debris.

Further purification of the proteins was achieved through reverse-phase high-performance liquid chromatography (RP-HPLC; Shimadzu, Kyoto, Japan) using an Intrada 5WP-RP column (Imtakt, Kyoto, Japan), with absorbance monitored at 220 nm. The mobile phases were Solution A (MilliQ-water + 0.1% trifluoroacetic acid (TFA)) and Solution B (acetonitrile + 0.05% TFA), with a flow rate of 1 mL/min and a column temperature of 30 °C. The analytical RP-HPLC purification was performed using acetic acid at a final concentration of 10% (*v*/*v*) and filtered through a 0.20 µm membrane filter to remove any aggregates. The purified RBD, with a purity greater than 95%, was lyophilized and stored at −30 °C.

### 4.2. Binding Activity by Bio-Layer Interferometry

The RBD binding affinity with hACE2 was measured using OCTET-N1 bio-layer interferometry (Sartorius, Goettingen, Germany). SARS-CoV-2 RBD (5 μg/mL) was immobilized on the Ni-NTA biosensor for 180 s, followed by an association phase with recombinant hACE2 (purity > 90% SDS-PAGE) (Bioworld Tech, St. Louis Park, MN, USA) diluted in kinetics buffer for 300 s. Dissociation was then carried out in the kinetics buffer for 300 s. Binding affinities were calculated using a 1:1 Langmuir binding model.

### 4.3. Mice Immunization

All mouse immunizations were performed according to the animal ethics guidelines and protocols set by the Tokyo University of Agriculture and Technology and the Japanese governmental regulations on animal experimentation. Two sets of immunization experiments were conducted using Jcl:ICR mice, all aged five weeks at the beginning of the experiment. One group (with a total of four mice) was immunized without adjuvant, while the other group (also with a total of four mice) was immunized in the presence of TiterMax Gold adjuvant (Sigma-Aldrich, St. Louis, MO, USA).

The RBD was dissolved in 10 mM Hepes buffer, pH 7.0, at a concentration of 30 µg per dose for immunization in the presence of an adjuvant. This solution was then supplemented with an equal volume of TiterMax Gold adjuvant (100 µL of protein plus 100 µL of adjuvant, totaling 200 µL per dose per mouse). One dose was administered subcutaneously to this group.

For immunization in the absence of an adjuvant, RBD was formulated in Hepes, pH 7.0, at a concentration of 30 µg per dose (100 µL per mouse). Three doses were injected subcutaneously at biweekly intervals, and the booster dose was given intraperitoneally at day 131. Additionally, one negative control mouse was injected with only Hepes buffer.

### 4.4. Anti-RBD IgG Titer by ELISA

The ELISA was performed to analyze the anti-RBD IgG titer. The 96-well 4HBX Immulon (ThermoFisher Scientific, Waltham, MA, USA) plates were coated with 2.5 µg/mL of purified Omicron BA.5 RBD at 4 °C for overnight. The following day, the plates were blocked with 1% BSA in PBS for 1 h at 37 °C. Mouse antisera were applied to the plates at the initial dilution of 1:1000, 1:2000, or 1:4000 in 0.1% BSA in PBS, followed by a 3-fold serial dilution, and plates were then incubated at 37 °C for 1 h. The plates were washed thrice with PBS-0.05% Tween-20. Finally, anti-mouse IgG HRP conjugate (Thermo Fisher Scientific, Waltham, MA, USA) was added at a 1:10,000 dilution in 0.1% BSA-PBS-Tween-20 and incubated for 1 h at 37 °C. Plates were washed and developed using OPD (o-phenylenediamine dihydrochloride) for 20 min at room temperature, and then 50 µL of 0.5 N sulfuric acid was added to stop the reaction. The chromatonic signal was measured at 492 nm using a microplate reader (SH9000 Lab, Hitachi High-Tech Science Co., Tokyo, Japan). Antibody titers were calculated from the power fitting of OD 492 nm versus the reciprocal of the antisera dilution, using a cutoff of OD 492 nm = 0.1 above the background value. The values were averaged over the number of mice (*n*) in the respective groups.

The binding affinity of antisera elicited by *E. coli*-expressed RBD to mammalian cell-expressed full-length spike protein (S1) was assessed by ELISA. The plates were coated with 4 µM of S1 (Acrobiosystems, Newark, DE, USA) or RBD in 1× PBS. Mouse antisera were then applied at a 1:1000 dilution, followed by the subsequent steps as mentioned above.

### 4.5. Cell Surface CD Marker Analysis

Splenocytes were extracted 141 days after the initial administration from the control mouse and two mice from each of the adjuvant and no adjuvant groups. The cells were then analyzed for cell surface CD markers using flow cytometry. Single-cell suspensions of mouse splenocytes were prepared in FACS (fluorescence activated cell sorter) buffer (PBS supplemented with 2% FBS (Fetal Bovine Serum), 1 mM EDTA, and 0.1% sodium azide). Subsequently, red blood cells (RBCs) were lysed using 1× RBD lysis buffer (0.15 M ammonium chloride, 10 mM potassium bicarbonate, and 0.1 mM EDTA).

Furthermore, one million splenocyte cells in 100 µL of pre-cooled FACS buffer were surface-stained with different fluorescence-labeled antibodies according to the manufacturer’s guidelines. To analyze CD4^+^ T-lymphocytes, cells were stained with anti-CD3-PCy5, CD4-PCy7, CD44-FITC, and CD62LPE-conjugated antibodies in one tube. For CD8^+^ T-lymphocytes, cells were stained with Anti-CD3-PCy5, CD8-PCy7, CD44-FITC, and CD62LPE-conjugated antibodies in another tube (0.2 µg of antibodies/100 µL) for 30 min in the dark. Unbound excess conjugated antibodies were removed by centrifugation, and the cells were resuspended in 500 µL of FACS buffer. The data were collected using a CytoFlex flow cytometer (Beckman Coulter, Brea, CA, USA).

### 4.6. ACE2 Binding Inhibition Assay

Hydrated Ni-NTA biosensors (Sartorius, Goettingen, Germany) were loaded with 5 μg/mL of SARS-CoV-2 Omicron BA.5 RBD for 480 s. The baseline interference phase was measured for 30 s in a kinetics buffer (KB: 1× PBS pH 7.4, 0.01% bovine serum albumin, and 0.005% Tween-20). Immunized mouse antisera at dilutions of 1:50, 1:100, 1:1000, and 1:10,000 were loaded for 200 s. Then the sensors were subjected to association phase immersion for 200 s in wells containing 400 nM recombinant hACE2 diluted in KB. Then, the sensors were immersed in KB for as long as 200 s in the dissociation step. The inhibition percent of ACE2 binding was calculated with respect to the buffer-immunized mouse antisera.

### 4.7. Pseudovirus Neutralization Assay

The neutralization assay was conducted using SARS-CoV-2 Spike (Omicron BA.4 and BA.5) Fluc Pseudovirus (Acrobiosystems, Newark, DE, USA). The human ACE2 293T cell line (TaKaRa Bio Inc., Shika, Japan) was cultured in DMEM supplemented with 10% FBS and 1% penicillin-streptomycin. Heat-inactivated antisera were diluted 1:1 and mixed with 25 µL of pseudovirus in a 96-well plate, followed by incubation at 37 °C in a 5% CO_2_ environment for 1 h. Subsequently, cells at a density of 4–5 × 10^5^ cells/mL were added to the wells at a volume of 100 µL and incubated for 48 h under the same conditions. After incubation, the medium was discarded, leaving approximately 100 µL in each well. Then, 100 µL of Britelite Plus reporter reagent (PerkinElmer, Norton, OH, USA) was added and incubated for 2 min at room temperature. Luminescence values (RLU) were measured using Varioskan LUX (ThermoFisher Scientific, Waltham, MA, USA).

The pseudovirus neutralization titer (ID_50_) was assessed by serial dilution of selected mouse antisera, mixed with 10 µL of pseudovirus, and seeded with 100 µL of cells at a density of 2–3 × 10^5^ cells/mL and incubated for 53 h. The subsequent steps were carried out as described above.

## 5. Conclusions

The robust immune response with long-term memory elicited by our *E. coli*-expressed RBD and the neutralizing efficacy of the antisera highlight its potential as a COVID-19 vaccine seed. Furthermore, the scalability, handiness, and adaptability of our *E. coli*-expressed RBD make it well-suited for addressing challenges posed by emerging variants. These findings emphasize the promising role of bacterial expression systems in vaccine development against COVID-19.

## Data Availability

The data presented in this study are available on request from the corresponding author. The data are not publicly available due to [Data sharing agreements with collaborators that limit public access].

## Supplementary Materials

The following supporting information can be downloaded at: https://www.mdpi.com/article/10.3390/molecules29112676/s1, Figure S1: Pseudo-virus inhibition fitting curve generated by the Quest Graph™ IC50 Calculator.
