# Supplementary material for: Non-Glycosylated SARS-CoV-2 Omicron BA.5 Receptor Binding Domain (RBD) with a Native-like Conformation Induces a Robust Immune Response with Potent Neutralization in a Mouse Model
Source: Molecules. 2024 Jun 5;29(11):2676. doi: 10.3390/molecules29112676 (PMC11173568; doi:10.3390/molecules29112676)
Supplement: Supplementary file 1 [file molecules-29-02676-s001.zip › molecules-3017218-supplementary.pdf]

Supplementary figure

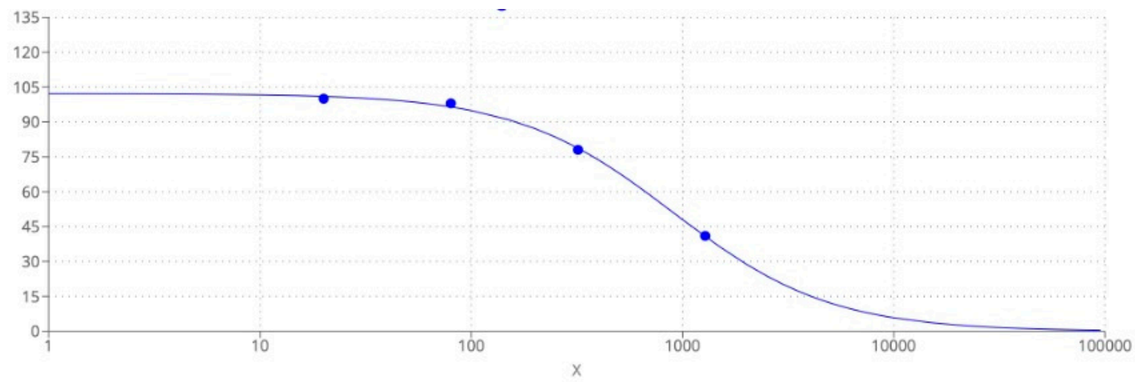

Figure S1: Pseudo-virus inhibition fitting curve generated by the Quest Graph™ IC50 Calculator.

ID_{50} was calculated using the following equation.

$$Y = \mathrm{Min} + \frac{Max - \mathrm{Min}}{1 + \left(\frac{X}{ID50}\right) hill\ coefficient}$$
